# Analysis of Mitochondrial DNA Polymorphisms in the Human Cell Lines HepaRG and SJCRH30

**DOI:** 10.3390/ijms20133245

**Published:** 2019-07-02

**Authors:** Matthew J. Young, Anitha D. Jayaprakash, Carolyn K. J. Young

**Affiliations:** 1 Department of Biochemistry and Molecular Biology, Southern Illinois University School of Medicine, Carbondale, Illinois 62901, USA; 2 Girihlet Inc., Oakland, CA 94609, USA

**Keywords:** human mitochondrial DNA (mtDNA), mtDNA homoplasmy, mtDNA heteroplasmy, cancer-derived cell lines, next-generation sequencing, Mseek, HepaRG, hepatocarcinoma, SJCRH30, rhabdomyosarcoma

## Abstract

The mitochondrial DNA (mtDNA) sequences of two commonly used human cell lines, HepaRG and SJCRH30, were determined. HepaRG originates from a liver tumor obtained from a patient with hepatocarcinoma and hepatitis C while SJCRH30 originates from a rhabdomyosarcoma patient tumor. In comparison to the revised Cambridge Reference Sequence, HepaRG and SJCRH30 mtDNA each contain 14 nucleotide variations. In addition to an insertion of a cytosine at position 315 (315insC), the mtDNA sequences from both cell types share six common polymorphisms. Heteroplasmic variants were identified in both cell types and included the identification of the 315insC mtDNA variant at 42 and 75% heteroplasmy in HepaRG and SJCRH30, respectively. Additionally, a novel heteroplasmic G13633A substitution in the HepaRG *ND5* gene was detected at 33%. Previously reported cancer-associated mtDNA variants T195C and T16519C were identified in SJCRH30, both at homoplasmy (100%), while HepaRG mtDNA harbors a known prostate cancer-associated T6253C substitution at near homoplasmy, 95%. Based on our sequencing analysis, HepaRG mtDNA is predicted to lie within haplogroup branch H15a1 while SJCRH30 mtDNA is predicted to localize to H27c. The catalog of polymorphisms and heteroplasmy reported here should prove useful for future investigations of mtDNA maintenance in HepaRG and SJCRH30 cell lines.

## 1. Introduction

More than 1000 human mitochondrial proteins are encoded by the nuclear genome and must be imported into mitochondria following translation on cytoplasmic ribosomes [1]. The mitochondrial DNA genome (mtDNA) is an ~16.6 kilobase pair (kbp) covalently closed circular molecule that contains 13 genes for polypeptides, 2 genes for rRNAs, and 22 genes for tRNAs [2,3,4]. Our maternally inherited mtDNA is critical to cellular viability as exemplified by the numerous disease mutations associated with it and by observations that knocking out mtDNA maintenance genes results in embryonic lethality in various mouse models [5,6,7]. Maintenance of the mitochondrial genome is also required to avoid apoptosis induced by mtDNA damage [8,9]. A single human cell can contain several thousand copies of mtDNA that are distributed within hundreds of individual mitochondria or throughout an elaborate mitochondrial reticular network [10,11,12]. Homoplasmy is the existence of clonal copies of mtDNA within a cell; however, mtDNA can also exist in heteroplasmy or a mixed population of different mtDNA genotypes within a cell [13,14]. Mitochondrial fusion and fission allow for complementation of heteroplasmic mtDNA genes in *trans* (e.g., a wild-type gene product complementing a mutant gene product); however, as the proportion of wild-type to mutant mtDNA decreases, the ability of a cell to produce energy via oxidative phosphorylation (OXPHOS) can decline [15]. Interestingly, enrichment of mtDNA heteroplasmic variants have been reported in certain cancers [16,17], and heteroplasmic variants have been identified in human peripheral blood mononuclear cells, 501T fibroblast cell lines, and cancer-derived cell lines [14,18].

Human cancer-derived cell lines are useful preclinical tools for studies of mitochondrial metabolism and cancer cell biology, as well as high-throughput drug toxicity and drug discovery experiments. SJCRH30 is a cell line that was derived from the tumor of a 17-year-old white male with rhabdomyosarcoma. SJCRH30 cells harbor attenuated sarcomere structures resembling those found in primitive rhabdomyoblasts [19]. SJCRH30 has been used to evaluate the cytotoxicity of chemotherapeutic drugs, such as cisplatin, doxorubicin, topotecan, and others [20,21]. Additionally, SJCRH30 has been used as a model of human myoblasts to study the regulation of mitochondrial biogenesis and cellular oxygen consumption rates [22,23]. 

HepaRG was originally derived from a liver tumor obtained from a female patient suffering from hepatitis C infection and hepatocarcinoma [24]. The HepaRG cell line expresses various mature hepatocyte-like functions, including cytochrome P450s associated with xenobiotic metabolism [25]. Importantly, HepaRG displays sensitivity to hepatotoxic compounds such as acetaminophen and aflatoxin B_1_ [25,26]. Recent studies support that HepaRG is a suitable model to test drug-induced mitochondrial toxicity and mtDNA homeostasis [3,26,27,28]. Here we report the mtDNA genome polymorphisms and heteroplasmy of SJCRH30 and HepaRG. 

## 2. Results

### 2.1. HepaRG mtDNA Nucleotide Variants and Heteroplasmy

The HepaRG mtDNA genome contains a single C at positions 3106–3107, which is in agreement with the revised Cambridge Reference Sequence, rCRS [29]. In contrast to the rCRS, HepaRG mtDNA contains 14 nucleotide variations, Table 1. With exception to the A16T transversion substitution and the insertion of C at position 315 (315insC), the remaining variants are transitions with A to G, G to A, T to C, or C to T changes. The MITOMASTER human mtDNA sequence analysis tool predicts HepaRG mtDNA localizes within haplogroup branch H15a1 [30]. H15a1 is of European origin [31]. 

Of the 14 variants, six have been previously reported as polymorphisms, A263G (non-coding), A750G (*RNR1*, 12 S ribosomal RNA), A1438G (*RNR1*), A4769G (synonymous substitution in the NADH dehydrogenase subunit 2 gene, *ND2*), A8860G (Thr112Ala missense in ATP synthase F0 subunit 6 gene, *ATP6*), and A15326G/Thr194Ala missense in the cytochrome b gene, *CYB* [18]. Additionally, the previously reported prostate cancer-associated T6253C missense substitution was detected in the HepaRG mtDNA genome [32]. The T6253C/Met117Thr substitution alters an evolutionarily conserved amino acid residue in cytochrome c oxidase subunit 1 (*COX1*) that could affect mitochondrial function as predicted by MutationAssessor [33]. MutationAssessor predicts the potential deleterious impact of a DNA mutation that changes a protein’s amino acid residue. To make this prediction, multiple sequence alignments of homologous proteins are grouped into families and subfamilies. Using the conservation pattern information generated from the alignments, MutationAssessor generates a functional impact (FI) score to rate a mutation’s impact as either high, medium, low, or neutral. The *COX1* Met117Thr variant has an FI score of 2.62 and is predicted to have a medium impact on protein function.

Three of the identified variants are not frequently associated with branch H15a1, A16T, 315insC, and G13633A. The non-coding A16T substitution is listed in the Single Nucleotide Polymorphism Database (dbSNP) as a single nucleotide variation (refSNP id: rs1556422363) and occurs at near homoplasmic levels, 94%. The 315insC insertion is located in the mtDNA control region and was detected at 42% heteroplasmy. To the best of our knowledge, the 33% heteroplasmic non-synonymous G13633A/Gly433Ser substitution localizing to the NADH dehydrogenase subunit 5 (*ND5*) gene has not been identified to date. The *ND5* G13633A/Gly433Ser mutation has an FI score of 2.55 and is predicted to have a medium impact on protein function. Therefore, the Gly433Ser variant could negatively affect mitochondrial function. Maintenance of HepaRG mtDNA heteroplasmy during growth in tissue culture was confirmed by sequencing at passages 11 (41% 315insC and 37% G13633A) and 16 (42% 315insC and 33% G13633A, Table 1). With the exception of the 315insC and the G13633A heteroplasmic variants, the remaining substitutions exist at ≥88% within the population of mtDNA molecules and are therefore near homoplasmic. The remaining mtDNA variants include two silent substitutions, C14953T and T11410C, as well as two non-coding substitutions, T55C and T57C. 

### 2.2. SJCRH30 mtDNA Nucleotide Variants and Heteroplasmy

Similar to the rCRS and to the HepaRG mtDNA sequences, the SJCRH30 mtDNA genome contains a single C at positions 3106–3107. SJCRH30 mtDNA contains 14 nucleotide variations relative to the rCRS, and MITOMASTER predicts localization within haplogroup branch H27c, Table 2.

The 315insC variant found in HepaRG mtDNA is also present in the SJCRH30 mitochondrial genome at 75% heteroplasmy. With the exception of 315insC, the remaining variants are transitions with A to G, G to A, or T to C changes. Six of the previously mentioned polymorphisms identified in HepaRG were detected in SJCRH30 mtDNA (A263G, A750G, A1438G, A4769G, A8860G, and A15326G). The common G11719A variant was also identified in SJCRH30 mtDNA [18]. Additionally, three of the nucleotide changes detected do not conventionally occur in H27c, namely T195C (non-coding), 315insC (non-coding), and the missense T14634C/Met14Val NADH dehydrogenase subunit 6 (*ND6*) variant. The *ND6* Met14Val substitution has an FI score of −0.075 and is predicted to be neutral via MutationAssessor analysis. The mtDNA T195C is a previously reported European Caucasian melanoma-associated substitution while T16519C has been reported to increase a woman’s risk of developing breast cancer or to be in linkage disequilibrium with a functional SNP that increases a woman’s risk [36,38]. With the exception of the heteroplasmic 315insC variant, the remaining mtDNA substitutions were homoplasmic or near homoplasmic. Three of the near homoplasmic mtDNA variants include the synonymous T4838C (91%) and the non-coding A16316G (90%) and G16129A (95%) variants. 

## 3. Discussion

Reduced mtDNA copy number and the presence of mtDNA mutations that alter OXPHOS have been reported to be common in cancer; however, functional mitochondria and mtDNA are necessary for cancer cell growth and tumorigenesis [15,17,39,40]. Human cancer cells harbor both homoplasmic and heteroplasmic mtDNA mutations, and cell culture experiments utilizing cancer cell lines have demonstrated that heteroplasmy can be stably maintained after many passages [14,16]. For these reasons, mtDNA polymorphisms and heteroplasmy were evaluated in two commonly used cancer-derived cell lines, HepaRG and SJCRH30. Previously reported cancer-associated mtDNA variants were found in both cell lines. HepaRG harbors the prostate cancer-associated T6253C substitution at near homoplasmy (95%) while SJCRH30 contains both the melanoma-associated T195C and the T16519C breast cancer-associated variants at homoplasmy [32,36,38]. According to the MITOMASTER database, both T16519C (SJCRH30, H27c) and T6253C (HepaRG, H15a1) occur at 100% in their respective haplogroup branches. To date, the T195C variant is not associated with the two representative H27c sequences found in the MITOMASTER database, Table 2. 

In HepaRG and SJCRH30 mtDNA, six previously reported polymorphisms were found to be homoplasmic (fixed) or nearly homoplasmic, occurring at ≥88% within the population of mtDNA molecules (Table 1 and Table 2). These polymorphisms appear at frequencies of ≥96.8% and of 100% in the H15a1 and H27c branches, respectively. The HepaRG mtDNA variants are maintained at near homoplasmy and occur at ≥88%, with the exception of 315insC (42% heteroplasmy) and G13633A (33% heteroplasmy), Table 1. Similarly, SJCRH30 mtDNA variants occur at ≥90% with the exception of the 315insC variant (75% heteroplasmy), Table 2. The 315insC insertion is found in the mtDNA control region while the G13633A substitution alters an evolutionarily conserved codon that may affect mitochondrial function. We hypothesize that the 315insC and the G13633A variants are under negative selection and that wild-type mtDNAs are being sustained to maintain functional mitochondria. In mitochondrial genomes from both cell types, many of the substitutions were near homoplasmy, ranging from 88% to 100% occurrence within the population of mtDNAs. We predict that the presence of near homoplasmy variants, e.g., synonymous substitutions, may expand to 100% homoplasmy during further passaging in cell culture. Moreover, a non-coding substitution or missense variant that does not negatively impact OXPHOS could expand to 100% homoplasmy. As the majority of the mtDNA nucleotide variants identified in both HepaRG and SJCRH30 are transitions, these substitutions likely arose from erroneous incorporation by the replicative mtDNA polymerase gamma or from deamination events as previously proposed [41,42,43]. The SJCRH30 T195C transition substitution is atypical for haplogroup branch H27c and localizes to the mtDNA control region heavy-strand origin of replication, which is important for initiation of mtDNA replication. T195C may have been subject to selection in melanoma and rhabdomyosarcoma to modify mtDNA replication and, by extension, mitochondrial metabolism. Numerous modifications in mitochondrial function have been implicated in cancer biology including shifting energy production, disrupting apoptosis signaling, increasing mutation of mtDNA, and altering antioxidant activity and reactive oxygen species production [39]. Interestingly, T195C was found to be significantly higher in melanoma patients in comparison to unrelated control individuals [36]. The HepaRG A16T transversion substitution lies within the non-coding 7S DNA control region and is not typical of the H15a1 haplogroup branch. One hypothesis is that 7S DNA can be utilized as a primer to initiate heavy-strand replication [44]. Perhaps the A16T 7S DNA modification was subject to selection in hepatocarcinoma to modify mtDNA replication initiation in cancer cells. Future next-generation sequencing studies of mtDNA from tumors and unaffected control tissues could determine whether T195C and A16T are rhabdomyosarcoma- and hepatocarcinoma-specific changes, respectively. 

## 4. Materials and Methods

### 4.1. Cell Culture

Proliferating HepaRG (Biopredic International Saint-Grégoire, France) were grown until passage 16, as previously described [24,26]. SJCRH30 cells (RC13, RMS 13, SJRH30, ATCC^®^ CRL­2061^TM^) were grown until passage 5 according to ATCC recommendations. Human cell lines were (1) cultured for the recommended number of passages to avoid marked phenotypic and morphological changes and not more than 20 passages if recommendations were not available, (2) screened for correct morphology and bacterial contamination via microscopy on days of feeding and passaging to ensure correct growth rates and appearances of cells, and (3) routinely screened for potential mycoplasma infection using in-house comparative PCR analysis with primers and controls kindly provided by Dr. Uphoff [45]. Dulbecco’s phosphate-buffered saline (DPBS)-washed cell pellets were frozen at −80 °C and then processed as described below. 

### 4.2. mtDNA Next-Generation Sequencing and Data Analysis

The Mseek method of sample processing and deep sequencing of mtDNA, as well as the procedure for data analysis, was conducted as previously reported [14]. Briefly, Mseek consists of (i) isolating total DNA from a thawed DPBS-washed cell pellet, (ii) digesting linear nuclear DNA (nDNA) with Exonuclease V, (iii) purifying the products using Ampure beads to remove short fragments, (iv) testing the results of the digestion with PCR primers specific for mtDNA and nDNA using 1 μl of the digested sample, (v) fragmenting the remaining sample using Covaris and end-repair, (vi) ligating barcoded adapters compatible with the sequencing platform to the fragments, (vii) amplifying the library utilizing universal adapters, and (viii) loading samples onto the Illumina NextSeq 500 platform. 

The sequencing data were generated as fastq files, as previously described [14]. Briefly, the sequences were filtered for quality (sequences with >10 consecutive nucleotides with *Q* < 20 were eliminated) and mapped to the revised Cambridge Reference Sequence (rCRS), accession NC_012920. Identical reads were identified as being clonal and were considered only once, irrespective of the number of copies, toward variant calling. A variant call was made only if there were at least three non-clonal reads carrying the variant, and a minimum coverage of 10 was required at the variant. Variants occurring on reads on one strand (with a skew greater than 0.1 or 10%) of the mtDNA were excluded to further reduce errors. The error rate in NextSeq reads are usually <1 in 1000 (phred score *Q* > 30) and requiring at least three non-clonal reads reduces the error rate to well under one in a million. Nuclear contamination was estimated using sequences that map to repeat elements such as long interspersed nuclear elements (LINEs) and short interspersed nuclear elements (SINEs), which only occur in nDNA. This enables a reliable estimation of the level of nDNA contamination. The fastq files have been submitted to the NCBI Sequence Read Archive (SRA) BioProject ID: PRJNA545541.

## 5. Conclusions

We determined the mtDNA sequence polymorphisms and heteroplasmy of two commonly used cell lines derived from patients with hepatocarcinoma (HepaRG) and rhabdomyosarcoma (SJCRH30). We expect that the catalog of polymorphisms and heteroplasmy reported here will prove useful for future investigations of mtDNA maintenance utilizing these cell lines. 

## Figures and Tables

**Table 1:** Sequence changes and heteroplasmy identified in HepaRG mitochondrial DNA (mtDNA).

MtDNA nt. Change ^a^	Location ^b^	Coverage ^c^	%Variant ^d^	%Freq in H15a1 ^e^	Remarks ^f^
A16T	CR: 7S	37,783	94	0	SNV
T55C	CR: 7S	57,845	90	80.65	SNV
T57C	CR: 7S, HVS2, O_H_57	57,847	90	61.29	SNV
A263G	CR: HVS2, O_H_	4,879	98	96.77	SNV ^g^
315insC	CR:HVS2, O_H_	5,392	42	4.84	Insertion of C
A750G	*RNR1*	48,180	88	100	SNV ^g^
A1438G	*RNR1*	70,033	95	100	Benign ^g^
A4769G	*ND2*/M100	14,223	90	96.77	Synonymous variant, ATA > ATG ^g^
T6253C	*COX1*/M117T	58,704	95	100	Missense variant, ATA > ACA; prostate cancer associated ^h^
A8860G	*ATP6*/T112A	39,614	95	96.77	Missense variant, ACA > GCA ^g^
T11410C	*ND4*/P217	28,048	92	100	Synonymous variant, CCT > CCC
G13633A	*ND5*/G433S	43,013	33	0	Missense variant, GGT > AGT
C14953T	*CYB*/I69	61,411	89	98.39	Synonymous variant, ATC > ATT
A15326G	*CYB*/T194A	62,038	93	100	Missense variant, ACA > GCA ^g^

^a^ Nucleotide (nt.) positions are numbered according to the revised Cambridge Reference Sequence (rCRS) light-strand, NC_012920.1. The rCRS was derived from a single person from haplogroup H2a2, see www.mitomap.org for details. The rCRS nucleotides are listed on the left-hand side of the nucleotide positions while detected changes (variants) are listed on the right-hand side. ^b^ The non-coding control region (CR) that contains the 7S DNA (7S), the hypervariable segment 2 (HVS2), the heavy-strand origins of replication (O_H_), and in HeLa, A549, 143B, and TK^−^ cancer-derived cell lines and immortalized lymphocytes the major replication origin at position 57, O_H_57 [34], https://www.mitomap.org/foswiki/bin/view/MITOMAP/GenomeLoci. ^c^ Coverage at the indicated position. ^d^ The percentage of the variant in the plus strand represents heteroplasmy; 100% represents homoplasmy. ^e^ MITOMASTER predicts that HepaRG mtDNA lies within the haplogroup branch H15a1 of European origin [30,31]; the MITOMASTER-predicted frequency of each variant within H15a is indicated and is based on a total of 62 H15a sequences. ^f^ SNV, single nucleotide variation as listed in NCBI dbSNP short genetic variations, https://www.ncbi.nlm.nih.gov/snp/; benign as listed in ClinVar, https://www.ncbi.nlm.nih.gov/clinvar/; synonymous and missense variant codon changes are shown. ^g^ These polymorphisms occur in most mtDNA genomes except for a small subcluster of haplogroup H that includes the rCRS [18]. ^h^ T6253C has previously been found to be associated with prostate cancer [32] and with primary open-angle glaucoma [35].

**Table 2:** Sequence changes and heteroplasmy identified in SJCRH30 mtDNA.

MtDNA nt. Change ^a^	Location ^b^	Coverage ^c^	%Variant ^d^	%Freq in H27c ^e^	Remarks ^f^
T195C	CR: HVS2, O_H_	127	100	0	Melanoma-associated ^g^
A263G	CR: HVS2, O_H_	60	100	100	SNV ^h^
315insC	CR: HVS2, O_H_	24	75	0	Insertion of C
A750G	*RNR1*	117	100	100	SNV ^h^
A1438G	*RNR1*	193	100	100	Benign ^h^
A4769G	*ND2*/M100	79	100	100	Synonymous variant, ATA > ATG ^h^
T4838C	*ND2*/P123	141	91	100	Synonymous variant, CCT > CCC
A8860G	*ATP6*/T112A	149	100	100	Missense variant, ACA > GCA ^h^
G11719A	*ND4*/G320	121	97	100	SNV, synonymous variant, GGG > GGA
T14634C	*ND6*/M14V	136	90	0	Missense variant, ATG > GTG ^i^
A15326G	*CYB*/T194A	129	95	100	Missense variant, ACA > GCA ^h^
G16129A	CR: HVS1, TAS2, 7S	154	95	100	SNV
A16316G	CR: HVS1, 7S	120	90	100	SNV
T16519C	CR: 7S	162	100	100	Associated with breast cancer risk ^j^

^a^ Nucleotide (nt.) changes are labeled as described in the footnote for Table 1; ^b^ The non-coding control region (CR) that contains the 7S DNA (7S), the hypervariable segments 1 and 2 (HVS1 and HVS2), the heavy-strand origins of replication (O_H_), and the extended termination-associated sequence (TAS2), https://www.mitomap.org/foswiki/bin/view/MITOMAP/GenomeLoci. ^c^ Coverage at the indicated position. ^d^ The percentage of the variant in the plus strand represents heteroplasmy; 100% represents homoplasmy. ^e^ MITOMASTER predicts that SJCRH30 mtDNA lies within the haplogroup branch H27c; the MITOMASTER-predicted frequency of each variant within H27c is indicated and is based on a total of two H27c sequences. ^f^ SNV, benign, and codon changes are as described in the footnote for Table 1; ^g^ T195C has been found to be associated with melanoma in European Caucasians [36] and bipolar disorder [37]. ^h^ These polymorphisms occur in most mtDNA genomes except for a small subcluster of haplogroup H that includes the rCRS [18]. ^i^ The heavy-strand is the *ND6* coding strand (the RNA is transcribed from, and could hybridize to, the light-strand). ^j^ T16519C was reported to increase a woman’s risk of developing breast cancer or is in linkage disequilibrium with a functional SNP that increases a woman’s risk [38].

## Abbreviations

mtDNA	mitochondrial DNA
rCRS	revised Cambridge Reference Sequence
OXPHOS	oxidative phosphorylation
kbp	kilobase pair
dbSNP	The Single Nucleotide Polymorphism Database
*ND5*	NADH dehydrogenase subunit 5 gene
